# Direct Observation of Defect Range and Evolution in Ion-Irradiated Single Crystalline Ni and Ni Binary Alloys

**DOI:** 10.1038/srep19994

**Published:** 2016-02-01

**Authors:** Chenyang Lu, Ke Jin, Laurent K. Béland, Feifei Zhang, Taini Yang, Liang Qiao, Yanwen Zhang, Hongbin Bei, Hans M. Christen, Roger E. Stoller, Lumin Wang

**Affiliations:** 1 Department of Nuclear Engineering and Radiological Science, University of Michigan, Ann Arbor, MI 48109-2104, USA; 2 Materials Science and Technology Division, Oak Ridge National Laboratory, Oak Ridge, TN 37831, USA; 3 Department of Materials Science & Engineering, University of Tennessee, Knoxville, TN 37996, USA; 4 Center for Nanophase Materials Sciences, Oak Ridge National Laboratory, Oak Ridge, TN 37831, USA; 5 School of Materials, The University of Manchester, Manchester M13 9PL, UK

## Abstract

Energetic ions have been widely used to evaluate the irradiation tolerance of structural materials for nuclear power applications and to modify material properties. It is important to understand the defect production, annihilation and migration mechanisms during and after collision cascades. In this study, single crystalline pure nickel metal and single-phase concentrated solid solution alloys of 50%Ni50%Co (NiCo) and 50%Ni50%Fe (NiFe) without apparent preexisting defect sinks were employed to study defect dynamics under ion irradiation. Both cross-sectional transmission electron microscopy characterization (TEM) and Rutherford backscattering spectrometry channeling (RBS-C) spectra show that the range of radiation-induced defect clusters far exceed the theoretically predicted depth in all materials after high-dose irradiation. Defects in nickel migrate faster than in NiCo and NiFe. Both vacancy-type stacking fault tetrahedra (SFT) and interstitial loops coexist in the same region, which is consistent with molecular dynamics simulations. Kinetic activation relaxation technique (k-ART) simulations for nickel showed that small vacancy clusters, such as di-vacancies and tri-vacancies, created by collision cascades are highly mobile, even at room temperature. The slower migration of defects in the alloy along with more localized energy dissipation of the displacement cascade may lead to enhanced radiation tolerance.

Ion beam irradiation has been extensively used as an efficient tool to evaluate the tolerance of materials to extreme radiation environments^1^,^2^,^3^,^4^,^5^,^6^. Moreover, ion beams have also been widely utilized to modify the physical and chemical properties of surfaces^7^,^8^,^9^,^10^. The interaction between energetic particles and the crystalline lattice of materials results in point defects and defect clusters, including dislocation loops, voids, and localized compositional changes^3^. The evolution of radiation-induced defect concentrations in materials can be described by three competing processes: (1) defect production from collision cascades; (2) subsequent vacancy-interstitial recombination within the diffusion volume; and (3) point defect absorption by different types of sinks such as dislocations, grain and phase boundaries^2^. The point defects that survive recombination and sink absorption may cluster leading to macroscopically observable degradation effects, such as irradiation hardening, embrittlement, void swelling and irradiation-induced creep. Numerous recent studies for developing radiation-tolerant materials, both theoretical and experimental, have focused on the introduction of high-density defect sinks to reduce residual defect concentrations, as in oxide-dispersion-strengthened steels (ODS)^11^,^12^,^13^,^14^,^15^ and nano-size polycrystalline alloys^16^,^17^. A comprehensive work based on CuNb multilayers, reported in detail by Demkowicz, shows that the layer interfaces are efficient sinks for absorbing irradiation induced point defects^18^. However, research focused on understanding how to control the defect dynamics at their early stages by enhancing defect recombination and controlling defect migration in single-phase alloys is still scarce, despite evidence that tuning the chemical composition of a single phase alloy may significantly change the features of defect clusters^19^,^20^.

Single-phase concentrated solid solution alloys (SP-CSAs) are a novel family of materials recently developed at Oak Ridge National Laboratory (ORNL) for studying defect dynamics without preexisting defect sinks. Unlike traditional alloy systems, SP-CSAs are composed of two to five principal elements that form random solid solutions in the simple underlying fcc or bcc lattices^21^,^22^,^23^,^24^. It is assumed that high-level lattice distortion and chemical complexity in SP-CSAs can substantially reduce the mean free path of electrons, phonons and magnons; which may modify defect formation energies and migration barriers. Therefore, SP-CSAs present a novel way to study defect stability, defect recombination, and defect migration mechanisms by tuning material chemical complexity. A comprehensive study of defect evolution in SP-CSAs would provide a fundamental understanding of radiation effects in metallic materials and yield new design principles for radiation-tolerant alloys.

In this study, pure nickel (Ni) and two SP-CSAs alloys of 50%Ni50%Co (at.%) and 50%Ni50%Fe (hereinafter termed as NiCo and NiFe) with the fcc structure were irradiated to various damage doses using charged gold (Au) ions. Comprehensive and direct observation of the defect structure, range, and evolution was accomplished using transmission electron microscopy (TEM). Rutherford backscattering spectrometry channeling (RBS-C) and computational simulations were also performed to complement the TEM measurements. The intrinsic properties of chemical complexity and the influence of specific elemental species on the distribution of defect clusters are discussed.

## Results

### Defect range in nickel, NiCo, and NiFe irradiated to various ion fluences

**Figure 1:**
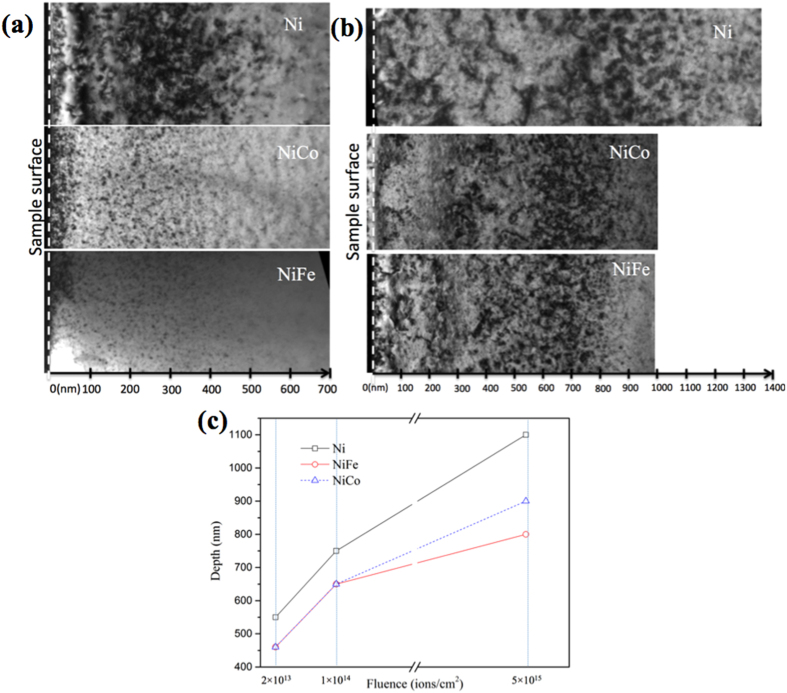
Defect distributions in nickel, NiCo, and NiFe after 3 MeV Au ion irradiation showing the damage range increases with increasing ion fluences and stretches deeper in nickel than in NiCo and NiFe. Bright-field cross-sectional TEM images (g = [200]) of the samples irradiated to (**a**) 2 × 10^13^/cm^2^ and (**b**) 5 × 10^15^/cm^2^. (**c**) Plots of the composition and dose dependence of the damage ranges in nickel, NiCo and NiFe irradiated by 3 MeV Au ions to 2 × 10^13^/cm^2^, 1 × 10^14^/cm^2^ and 5 × 10^15^/cm^2^.

Figure 1(a) shows panoramic cross-sectional TEM images of nickel, NiCo, and NiFe irradiated by 3 MeV Au ions at room temperature to a fluence of 2 × 10^13^/cm^2^ with a peak damage level of ~0.12 displacement per atom (dpa). The depth profile of displacement damage was predicted using the Stopping and Range of Ions in Matter (SRIM) code with the Kinchin-Pease option^25^ assuming a displacement threshold energy of 40 eV^26^ (see Supplementary Figure S1). The irradiation was carried out at an angle of 7° from the surface ([001] direction) to minimize channeling effects. Cross-sectional TEM analysis is important for studying the damage distribution beneath the sample surface of irradiated material, because damage introduced by ion irradiation is depth dependent. TEM samples were all prepared using focused ion beam (FIB) milling followed by a “flash polishing” technique that effectively reduced the damage introduced by FIB thinning (Figure S2). Bright-field (BF) TEM images were taken under two-beam conditions at g = [200]. High-density black dots, manifesting irradiation-induced defect clusters, were observed from the sample surface to an extended depth. At this initial fluence, the radiation-induced damage in NiCo and NiFe stretched to about 460 nm from the surface, which is consistent with the SRIM predictions. However, a wider damage region was observed in pure nickel, stretching to 550 nm in depth. The extended damage range indicates that the defect or defect clusters migrated deeper in pure nickel than in the two alloys even though, according to the SRIM simulations, the energetic Au ions should stop at a similar depth in all three materials.

Figure 1(b) shows the cross-sectional TEM images of the three materials irradiated to a much higher ion fluence (5 × 10^15^/cm^2^) with a peak displacement damage level of ~30 dpa. The existing damage bands extended much deeper from the surface after this higher fluence. The defect clusters stretched to a depth of ~1,100 nm in pure nickel but was limited to about 900 and 800 nm in NiCo and NiFe, respectively. An intermediate fluence (1 × 10^14^/cm^2^) with a peak damage level of ~ 0.6 dpa was also investigated, and the observation as a function of ion fluence is presented and discussed below.

Dependence of damage range on ion fluence in nickel, NiCo and NiFe samples is plotted in Fig. 1(c), based on the cross-sectional TEM characterization. The plot clearly shows two trends. First, in all three materials, the range of defect distribution beneath the irradiated surface increased dramatically with increasing ion fluence. The range of visible damage almost doubled when the irradiation fluence increased from 2 × 10^13^ to 5 × 10^15^/cm^2^, far exceeding the SRIM predicted depth even after taking into account the increased damage level at the tail of the damage profile under the high ion fluence. Second, the composition of the material seems to have a great impact on defect distribution, suggesting a very different defect migration property among the three materials. It is clear that the defects in nickel stretched much deeper than in the two binary alloys, indicating a higher defect migration rate in nickel; this observation stands true for both high and low dose irradiation.

**Figure 2:**
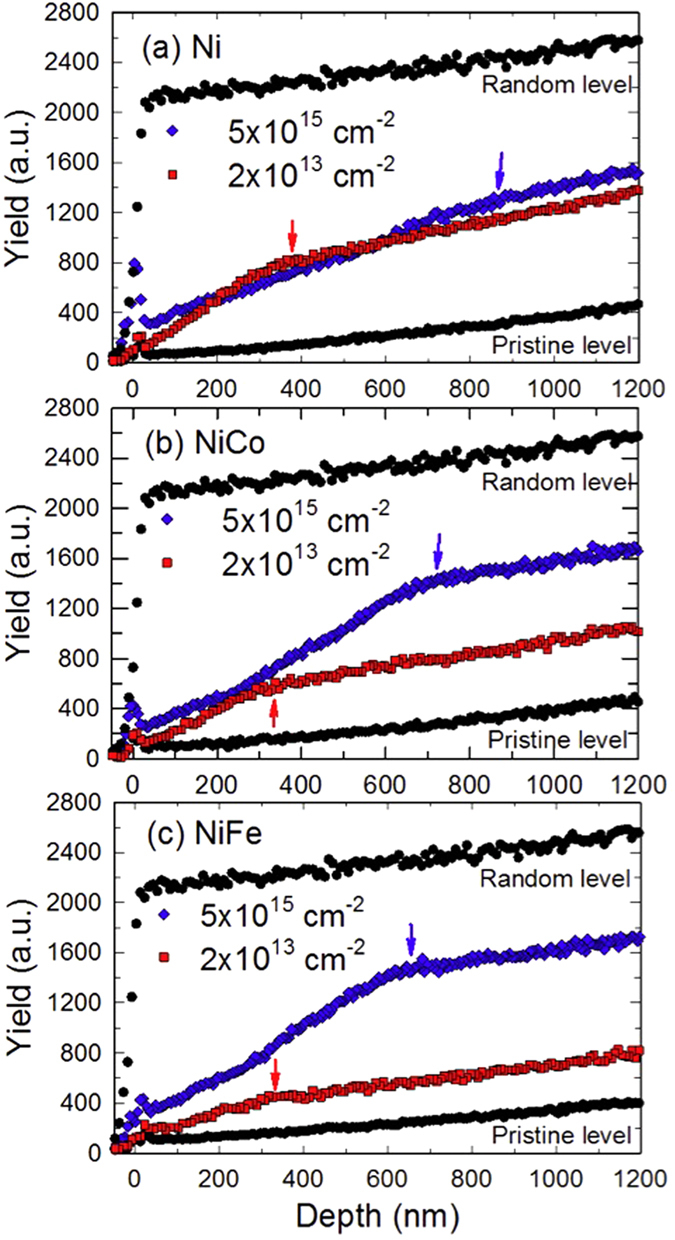
RBS-C spectra showing depth distribution of damage with the same trend as revealed in the TEM observation. (**a**) nickel, (**b**) NiCo, and (**c**) NiFe after irradiation by 3 MeV Au ions to 2 × 10^13^ and 5 × 10^15^/cm^2^.

Figure 2 shows the RBS channeling (RBS-C) spectra from irradiated (a) nickel, (b) NiCo, and (c) NiFe using 3 MeV Au ions at two fluences, 2 × 10^13^ and 5 × 10^15^/cm^2^, measured with 3.5 MeV He ions. In RBS-C measurements for metals (assuming dislocations and stacking faults are the major defect types), the derivative of the channeling yield, as a crude approximation, can be seen as an estimate of the overall lattice distortion, which contributes to the dechanneling of incident He ions^27^,^28^. The “knee” points to where the derivative goes down to a “pristine” level (marked with arrows) in each channeling curve of Fig. 2, which approximately indicates the depth corresponding to the end of the damage range^28^,^29^,^30^. The damage range for the high-fluence irradiation (5 × 10^15^/cm^2^) in all three materials is significantly larger than that for the low-fluence irradiation (2 × 10^13^/cm^2^) according to the positions of the “knee” points. Furthermore, it is very clear that the damage range in nickel is much deeper than that in NiCo and NiFe after the high fluence irradiation, while the difference in range is less significant after the low fluence irradiation. Compared with the TEM observations, the damage ranges measured by RBS-C are slightly shallower in each sample. For instance, the damage ranges in NiCo irradiated to 2 × 10^13^ and 5 × 10^15^/cm^2^ measured by RBS-C are about 350 and 700 nm, respectively, whereas the damage ranges observed in TEM are 460 and 900 nm under the two fluences. It is worth noting that, in the RBS-C measurements, the depths are calculated based on the density of the un-irradiated sample. In other words, possible irradiation-induced volume swelling is not taken into consideration in the RBS analysis. As a result, it is reasonable to expect that the depth measured by TEM might be larger than the ion channeling results shown here. In addition, the use of “knee” points in the determination of damage range is based on several assumptions and approximations, and the criteria for the “end” of damage range may not be exactly the same for the two techniques of characterization. Nevertheless, data from TEM and RBS-C do qualitatively support the same conclusion, i.e., suggesting the same damage range dependence on ion fluence and material composition.

### Defect evolution in NiFe

**Figure 3:**
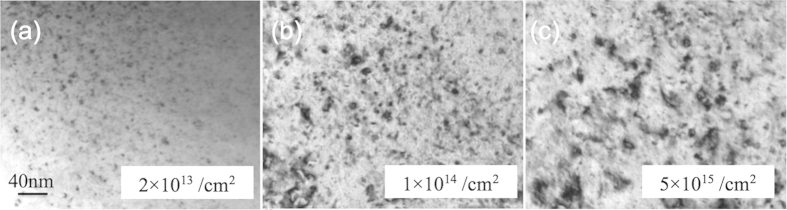
Defect clusters in NiFe grow significantly with increasing Au ion fluence. After (**a**) 2 × 10^13^/cm^2^, (**b**) 1 × 10^14^/cm^2^, (**c**) 5 × 10^15^/cm^2^.

Figure 3 shows the morphological evolution of defect clusters in NiFe with the ion fluences up to 5 × 10^15^/cm^2^. The regions with the highest defect density were chosen for the comparison, again using the two-beam diffraction condition with g = [200]. It is clear that at a lower dose, most of the defect clusters are in the form of small black dots with an average size < 4 nm. The black-dot defect clusters grew and transformed into distinguishable dislocation loops with the increased ion dose. Larger loops (up to ~17 nm in dimension) were found in NiFe when the fluence reached 5 × 10^15^/cm^2^, while there were still many small black-dot defects remaining in the matrix. A similar trend on defect cluster growth with increased ion fluence was found in pure nickel and NiCo.

### Nature of radiation-induced defect clusters in nickel and the two binary alloys

**Figure 4:**
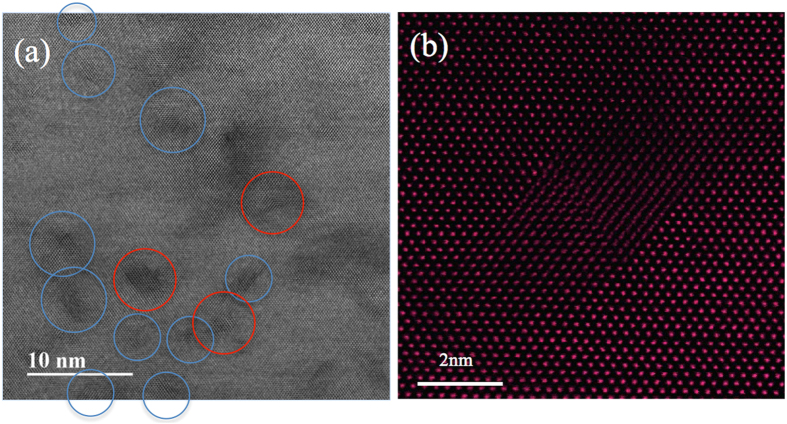
STEM images of SFT in NiFe irradiated by 3 MeV Au ions to 5 × 10^15^/cm^2^. (**a**) BF STEM image showing SFTs either distributed separately (blue circle) or coupled to form parallelograms (red circle); (**b**) High-resolution HAADF image showing a parallelogram made by two SFTs. The images were taken with the electron beam direction parallel to [100].

Nano-size stacking-fault tetrahedra (SFT) have been identified in all three irradiated materials under TEM at high magnification. Figure 4(a) shows the typical morphology of SFT in NiFe irradiated to 5 × 10^15^/cm^2^. The image was obtained by high-resolution scanning TEM (HR-STEM) with the foil oriented with the [011] zone axis parallel to the electron beam. The SFT are visible as open triangles bordered by {111} planes. Most of the SFT are dispersed in the matrix (blue circles), but some SFT coupled with one another to form a parallelogram (red circle). Figure 4(b) presents a clearer parallelogram feature at a higher magnification; the image was taken with a high-angle annual dark field (HAADF) detector in the STEM mode. Based on the features in the images of the SFT and the previous analysis from Jenkins, the hypothesis is that the SFT found in this study are all vacancy-type clusters^31^.

**Figure 5:**
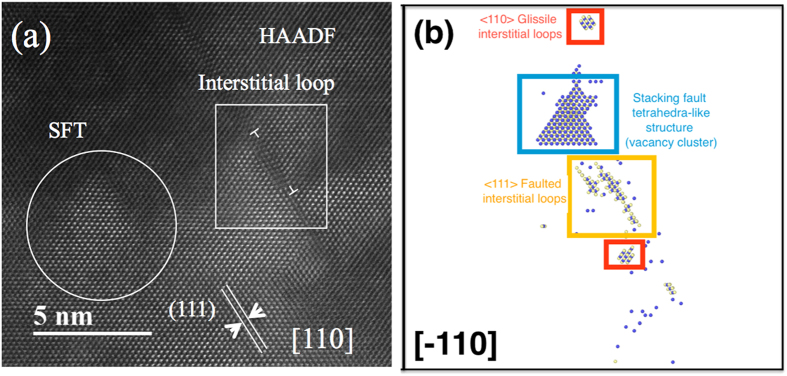
HR-HAADF STEM image and result from MD simulation, both showing vacancy-type SFT and interstitial dislocation loops co-exist in irradiated nickel. (**a**) HAADF image; (**b**) result from MD simulation.

Figure 5(a) shows an edge-on image of a 5 nm diameter dislocation loop lying on a {111} plane. The existence of the extra plane (indicated by the “T” signs in the image) is clear evidence of an interstitial loop with b = 1/3 <111> and is adjacent to a small stacking fault tetrahedron (in the white circle). Figure 5(b) is a snapshot from a molecular dynamics (MD) simulation of a displacement cascade in nickel caused by a 40 keV primary knock-on atom, using the 2013 Bonny potential^32^. Blue spheres represent the vacancies and beige spheres represent the interstitials. A <111> faulted interstitial loop is seen near a vacancy-type SFT-like structure, which is in very good agreement with the TEM observation shown in Fig. 5(a). Interstitial-type dislocation loops were observed in all samples, but no vacancy type of loops were found in either the TEM observation or the MD simulation. Kinetic activation relaxation technique (k-ART) simulation also shows that vacancies prefer to accumulate into SFT rather than loops (a detailed discussion will be presented in the discussion section below). Based on previous MD simulations of copper^33^,^34^, it is believed that small vacancy loops are unstable and transform immediately into vacancy-type SFT. Thus we assume most of the irradiation-induced vacancies that survived recombination clustered into SFT, and the surviving interstitials condensed into dislocation loops.

## Discussion

SRIM simulations were used to estimate the depth distribution of incident ion and displacement damage in the targets^35^. It is well know that incident ion mass, energy, as well as target composition and density are the dominant parameters controlling the range of primary damage. The significant dependence of damage range on ion fluence observed in this study implies that the important mechanisms of defect migration and damage accumulation are not predicted by the SRIM simulations. In addition, the large difference in the visible damage range between nickel and the two alloys (NiCo and NiFe), especially after the high ion fluence, cannot be explained by the SRIM simulations.

Friedland^36^ and others noted a large difference between ion irradiation–induced damage depth and theoretically simulated range. Extraordinary deviation of the damage depth from the theoretical prediction was found in pure Cu^36^,^37^,^38^, Mo^30^, Pt^39^, and Fe^40^ by α-particle channeling in a backscattering geometry. Changes in microhardness and wear resistance properties at much deeper depths than the projected ion range were noted^41^. These changes in the material properties far beyond the ion range were considered to be the result of dislocation migration in the irradiated materials. Direct evidence of this phenomenon has been reported by Sharkeev^42^,^43^ and his co-workers using TEM characterization. It was reported that a change in the dislocation structures might stretch to a depth of tens of microns even when the predicted ion range was only a few hundred nanometers. The phenomenon is named “long-range effect”. However, because of the limitations of the plan view TEM sample preparation technique used by these researchers, the error bar on the depth estimation was as large as 1 μm, and depth dependence of defect distribution in the whole range could not be observed. The extended damage range has also been observed in Ar ion irradiated Si based on the measurement of positron annihilation spectroscopy^44^ and Au ions irradiated magnesium oxide (MgO) measured by RBS-C^45^. In this study, direct observation of defect migration and the long-range effect were demonstrated clearly by cross-sectional TEM characterization.

The difference between the SRIM simulations and the experimental data on damage range can be explained by the influence of kinetic processes that take place over much longer times than the cascade damage events. The SRIM model does not account for defect migration and aggregation that give rise to the visible damage, which is at times much longer than the initial collisions. Thus, damage range calculated by SRIM does not depend on ion fluence. Based on the results of this study and previous research documented in literature, it is believed that the dependence of damage range on ion dose and material composition for pure nickel, NiCo, and NiFe may arise from the following mechanisms.

### Migration of point defects (interstitials/vacancies) and defect clusters

**Figure 6:**
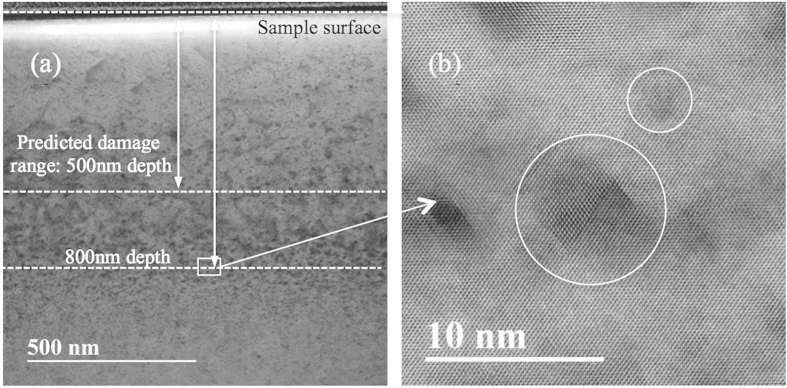
SFT were found far beyond the predicted damage range. (**a**) Cross-sectional STEM-BF image in NiFe irradiated by 3 MeV Au ions to a fluence of 5 × 10^15^/cm^2^; (**b**) HR-STEM image of SFT.

It is generally believed that interstitials are very mobile but vacancies are sessile at room temperature because of the high activation energy required (~1.09 eV) for vacancy migration. However, vacancy-type SFT were observed both inside and outside cascade formation regions in our study. Figure 6(a) shows the cross-sectional bright- field STEM image from a NiFe irradiated by 3 MeV Au ions to a fluence of 5 × 10^15^/cm^2^. The predicted damage range was only about 500 nm from the sample surface, as shown in the SRIM profile in Supplementary Figure S1. However, SFT were also found at 800 nm, as shown in Fig. 6(b). To understand this, an evolution of displacement cascade debris in nickel was studied using k-ART^46^,^47^,^48^. Animations of two typical k-ART simulations are included in Supplementary Video S1. One example is shown at 300 K (room temperature) and another at 900 K. At 300 K, interstitials (blue) are observed to diffuse and aggregate/annihilate, followed by the diffusion of di-vacancy and tri-vacancy clusters (red). One eventually annihilates with a nearby interstitial cluster. These vacancy clusters diffuse with a migration barrier of 0.3–0.4 eV, which corresponds to microsecond waiting times at room temperature, assuming a standard 10 THz attempt frequency. At 900 K, interstitial and vacancy clusters diffuse on time scales shorter than a nanosecond. In that particular example, vacancies form a small cluster with a SFT-like structure (6 + 1 vacancies) that is highly mobile. The activation barrier for this diffusion is of the order of 0.8 eV, which corresponds to a few milliseconds at room temperature. In both cases, a few interstitials that would otherwise have escaped the cascade volume, reentered because of the periodic boundaries and then reacted with the cascade debris.

The k-ART simulations provide crucial insights into the evolution of the defects generated by the displacement cascade events. Aggregation and annihilation both play a role, as well as diffusion of defects and defect clusters out of the volume affected by the displacement cascade. Notably, even though single vacancies are not mobile at room temperature, cascades create a significant number of vacancies close to one another, which can form small clusters. These vacancy clusters are significantly more mobile than single vacancies. The high mobility of self-interstitials and small vacancy clusters may help explain the presence of loops and SFT deep in the irradiated samples. This analysis is largely in agreement with the findings of Wang *et al.*^49^ who performed kinetic Monte Carlo (kMC) simulations in copper using transition states generated with ART-*nouveau* (ARTn), combined with insights provided by MD simulations. They found that the initial stages of irradiation damage recovery was explained by the diffusion of these small vacancy clusters. This is also consistent with simulations of small tetrahedral vacancy clusters in copper, which were shown to have higher mobility than single vacancies^50^ and to have the potential to react and form SFT. It is also proposed that stress gradients introduced by the implanted interstitials might boost the diffusion rate of single vacancies, with compressive stress shown to lower activation barriers for vacancy diffusion in bcc iron^51^.

Based on the above discussion, the formation of dislocation loops and SFT beyond the projected range may be aided by both point defect and defect cluster migration. The prolonged irradiation period (with increasing doses) will lead the point defect and defect clusters to migrate further. On the other hand, the MD simulations in a previous study show that the kinetics of defect cluster formation is considerably slower in NiFe than in pure nickel, indicating that defect migration barriers are higher in alloys than pure nickel^52^. In the other words, the defects have a higher diffusivity in nickel than in NiFe, which might be used to explain the differences in the damage range between pure nickel and the two nickel alloys.

### Effects of mechanical stress from implanted impurities

Because defects may migrate in all directions from where they were produced, enhanced defect migration alone cannot explain the extended depth of damage range observed at higher ion fluences. A high level of mechanical stress caused by ion implantation in the depth region where the ions stop may assist defect propagation into the bulk material in the direction perpendicular to the sample surface. This can result in an extended damage range with enhanced defect migration with increased ion fluence. High-concentration impurities (Au ions) implanted in this study would undoubtedly cause significant mechanical stress in the irradiated materials. High-level mechanical stress in ion irradiated areas of the material have been measured by laser interferometry and also confirmed by theoretical calculations^53^,^54^ in previous publications. X-ray diffraction measurements were performed on samples irradiated to very high fluence (1.1 × 10^17^/cm^2^) in order to investigate possible irradiation-induced stress. The results indicate that such high-fluence irradiations have induced a high-level mechanical stress (Supplementary: Stress study) in Au ion irradiated NiCo and NiFe. The high-level stress due to ion fluence of 10^17^/cm^2^ suggests possible low-level stress in the irradiated samples resulting from low-fluence irradiation. Point defects can diffuse from the projected range into deeper regions of the material and subsequently generate new dislocation loops under the stress gradient. In addition, Yoshitaka stated that defect clusters could exhibit fast migration in materials^55^, so the dislocation loops in the projected range also have the potential to move to a greater depth^42^. Higher ion fluence irradiation leads to a higher stress field gradient in the material because more Au ions are implanted into the sample. It is also worth noting that, with a constant ion flux, a more prolonged time is required for the high-fluence irradiation, e.g., 250 times longer irradiation time to reach 5 × 10^15^/cm^2^ than that the case for the low-fluence irradiation (2 × 10^13^/cm^2^). As a result, the migration of the point defects and dislocation loops may be further enhanced during the high-fluence irradiation than that under the low-fluence irradiation. The range of the defective layers in nickel, NiCo, and NiFe, irradiated at a low fluence of 2 × 10^13^/cm^2^, shown in Fig. 1(c), matches well with the SRIM predicted range. However, after higher-dose irradiation (1 × 10^14^ and 5 × 10^15^/cm^2^), the defect clusters stretch to a much greater depth because of the prolonged period needed for defects to migrate, in addition to the increased mechanical stress from the higher concentration of implanted interstitial impurities (Au ions in this case).

To evaluate the possible strain induced from the incorporation of Au atoms, we performed two additional irradiations at an extremely high fluence of 1.1 × 10^17^/cm^2^ in the NiCo and NiFe samples. The Au concentration at the peak of the Au profile predicted by SRIM is ~ 7%. Ion channeling measurements were performed to determine the location of the Au atoms, and the results indicate that all the implanted Au atoms occupy lattice sites, rather than interstitial sites.

When comparing the cross-sectional TEM images in Fig. 1(a), it is noteworthy that the defect clusters are distributed throughout the damage bands, except near the surface. Since the highest stress accumulates at the center of the implanted layer, the irradiation-induced stress should drive defect clusters to migrate both to the surface and to greater depths beyond the irradiated layer. Two potential explanations for seeing fewer defect clusters near the surface region are considered: (1) The defects indeed migrated to the near-surface region but subsequently escaped from the surface; or (2) the forward ion momentum suppressed defect migration to the surface during continuous implantation.

The final observed defect distributions are the integrated results of defect production, recombination, migration and clustering. Chemical complexity in a disordered solid solution can modify and tune the energy dissipation/transport of the irradiation through electrons and phonons, subsequently impacting the defect formation and evolution. A recent study shows that both electron mean free path and electrical thermal conductivity could be greatly decreased with increasing chemical complexity^26^. Thermal spike along the ion path and in the cascades can be prolonged because of the localized electron-electron interactions. Therefore, the defect dynamics have been significantly modified to suppress damage accumulation in SP-CSAs^26^. MD simulation shows that defect migration barriers are higher in SP-CSAs than that in pure nickel because of the intrinsic chemical disorder and lattice distortion^52^. The results of this study confirm the MD predictions that point defects/defect clusters migrate much slower in SP-CSAs than in pure nickel. Based on these results, it is plausible to speculate that the defect recombination rate would increase because the production and migration of defects are confined to a more localized area in SP-CSAs than in pure nickel. Therefore, SP-CSAs may demonstrate higher radiation tolerance under harsher radiation conditions.

The observed defect clusters migrated deeper in NiCo than in NiFe when the ion fluence increased to 5 × 10^15^/cm^2^, indicating that varying the atomic species of the alloys has a significant impact on defect evolution in SP-CSAs. The chemical species of the SP-CSA alloys on defect migration should be studied in further detail, particularly by computer simulation.

In summary, a synergistic study has been conducted on defect range and evolution in ion-irradiated single crystalline nickel and two Ni-based binary alloys, using cross-sectional TEM, RBS-C, MD and k-ART simulations. The results indicate that defect clusters distributed to a depth far exceeding the theoretically predicted damage range at the high ion fluence, and that the damage range in nickel is wider than in the alloys. High-diffusivity of the defects and prolonged irradiation period both contribute to this long-range effect observed after the high ion fluence. Mechanical stress created by implanted species may also play an important role in driving the defects to a deeper depth. It is believed that composition complexity has a significant impact on energy dissipation and defect dynamics, leading to a different migration rate of defects in nickel, NiCo, and NiFe. This study validates the simulation result^52^, which predicted that defects migrate much faster in nickel than in NiCo and NiFe. The production and migration of defects to a more confined local area in the alloys than that in the pure nickel may contribute to an enhanced defect recombination rate, leading to a higher radiation tolerance of the material.

## Methods

### Au ion irradiation

Mechanically polished nickel, NiCo, and NiFe were irradiated with 3 MeV Au ions to fluences of up to 2 × 10^13^/cm^2^, ~1 × 10^14^/cm^2^, and ~5 × 10^15^/cm^2^ at room temperature in the Ion Beam Materials Laboratory at the University of Tennessee. Two high-fluence irradiations of 1.1 × 10^17^/cm^2^ were performed in NiCo, and NiFe. A raster beam was used to create a homogeneous implantation. Predictions of local dose and ion stopping range in samples were calculated by the SRIM 2013 code in Kinchin-Pease option simulation mode assuming a displacement threshold energy of 40 eV, as shown in Supplementary Figure S1.

### Microstructural characterization

TEM and STEM samples were all prepared by FIB lift-out techniques using an FEI Helios Nanolab Dualbeam workstation. A flash polishing technique was developed to remove FIB-induced damage that might easily overlap with the damage from the primary ion irradiation. The standard lift-out procedure for cross-sectional TEM sample preparation was first conducted by FIB. Initial polishing used a 30 kV Ga^ + ^beam with a ± 1.5° angle between the Ga^ + ^beam and the sample surface. Final thinning and cleaning were performed at 5 kV with an incidence angle of ±7° for 5 min. The flash polishing was done using an electropolishing apparatus with an electrolytic solution of 4% perchloric acid in 96% ethanol. An electric potential of 6~14 V was applied to the FIB-polished specimen at a temperature range between −30 and −50 °C. The polishing period was controlled between 0.05 and 0.2 s with an accurate timer. The result of this technique is shown in Supplementary Figure S2, which shows the bright-field cross-sectional TEM images of the NiCo sample before and after flash polishing following the lift-out procedure using standard FIB techniques. A double Cs-corrected STEM (JEOL 3100R05) was employed for STEM imaging with an inner angle of 59 mrad and camera length of 15 cm. The image pixels and exposure time/pixel for imaging in the experiment were 1024 × 1024 and 30 μs. Bright-field imaging was performed with a JEOL 3011 TEM operated at 300 keV. Images were taken at an exposure time of 2 s.

### RBS channeling (RBS-C)

The ion channeling technique is an effective analytical technique for detecting damage distribution along certain crystalline orientations of irradiated samples. When ions move along the low-index lattice axes or planes of a single crystal, they experience smaller close-encounter probability and thus have less chance of being backscattered, compared with travelling in amorphous materials or in “random” directions. However, when the crystal is distorted, the backscatter probability will increase (dechanneling effect), and as a result, RBS-C can be used to study lattice disorder by measuring the backscattering yield. In addition, with known stopping power, RBS-C can give the depth where the backscattering occurs. Thus, RBS-C channeling is able to provide the depth-resolved damage profile, which can serve as a complementary technique to the TEM observations.

In this RBS-C study, pre-damaged nickel, NiCo, and NiFe were characterized by RBS-C along the [100] direction using 3.5 MeV He^+^ ions.

### Kinetic activation relaxation technique (k-ART)

The evolution of displacement cascade debris in nickel was studied using k-ART (as shown in Supplementary Video S1)—a fully atomistic, off-lattice, on-the-fly kMC algorithm^46^,^47^,^48^. At every time step, events were generated using ARTn^56^, and the residence-time algorithm determined the time evolution^57^. This method was shown to accurately simulate the time evolution of irradiation damage in crystalline silicon^58^,^59^ and bcc iron^51^ over time scales comparable to experiment. The implementation of k-ART used in this study employed the active volume concept^51^,^60^ to reduce the computational cost of the simulations. Initial configurations for k-ART simulation were obtained by performing simulations of 10 keV displacement cascades in a 256,000-atom box using MD. k-ART simulations were performed at 300 K and 900 K, with the simulated time reaching as long as a few seconds. Nickel interatomic interactions were modeled using the 2013 Bonny potential^32^.

### Molecular dynamics (MD)

MD simulation in nickel was performed using the LAMMPS software package^61^. Interatomic interactions were described using the 2013 Bonny potential^32^. Displacement cascades were simulated, giving the primary knock-on atom kinetic energy in the [135] direction, at 40 keV energies. The MD simulation was run at a constant NVE (256,000 atoms, equilibrated at room temperature and 0 pressure) and a variable time-step limited the distance traveled by atoms in each step. Runs were made with and without a Langevin bath at 300 K. The presence of the Langevin bath had no significant effect on the outcome. The analysis of defects was performed using Wigner-Seitz cell analysis and the equivalent sphere analysis.

## Additional Information

**How to cite this article**: Lu, C. *et al.* Direct Observation of Defect Range and Evolution in Ion-Irradiated Single Crystalline Ni and Ni Binary Alloys. *Sci. Rep.* **6**, 19994; doi: 10.1038/srep19994 (2016).

## Supplementary Material

Supplementary Information

Supplementary Video S1(a)

Supplementary Video S1(b)
