# Supplementary material for: Direct Observation of Defect Range and Evolution in Ion-Irradiated Single Crystalline Ni and Ni Binary Alloys
Source: Sci Rep. 2016 Feb 1;6:19994. doi: 10.1038/srep19994 (PMC4734288; doi:10.1038/srep19994)
Supplement: Supplementary Information [file srep19994-s1.pdf]

Direct Observation of Defect Range and Evolution in Ion-Irradiated Single Crystalline Ni and Ni Binary Alloys

Chenyang Lu[1*], Ke Jin[2,3], Laurent K. Béland[2], Feifei Zhang[1], Taini Yang[1], Liang Qiao[4,5], Yanwen Zhang[2], Hongbin Bei[2], Hans M. Christen[4], Roger E. Stoller[2], Lumin Wang[1*]

[1]Department of Nuclear Engineering and Radiological Science, University of Michigan, Ann Arbor, MI 48109-2104, USA
[2]Materials Science and Technology Division, Oak Ridge National Laboratory, Oak Ridge, TN 37831, USA
[3]Department of Materials Science & Engineering, University of Tennessee, Knoxville, TN 37996, USA
[4]Center for Nanophase Materials Sciences, Oak Ridge National Laboratory, Oak Ridge, TN 37831, USA
[5]School of Materials, The University of Manchester, Manchester M13 9PL, UK
**Corresponding authors: chenylu@umich.edu; lmwang@umich.edu*

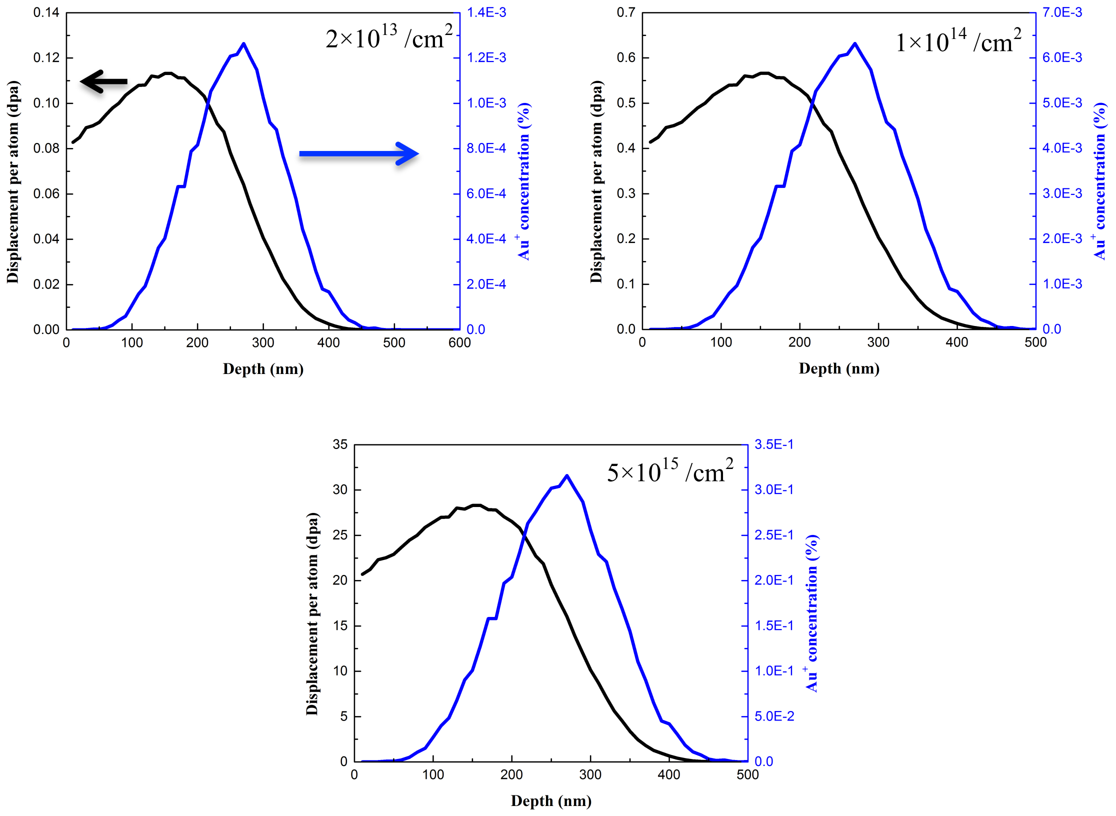

Supplementary Figure S1. Predicted distributions of damage and Au concentration in pure Ni irradiated by 3 MeV Au ions to a fluence of (a) 2×10^{13} /cm^2; (b) 1×10^{14} /cm^2; (c) 5×10^{15} /cm^2.

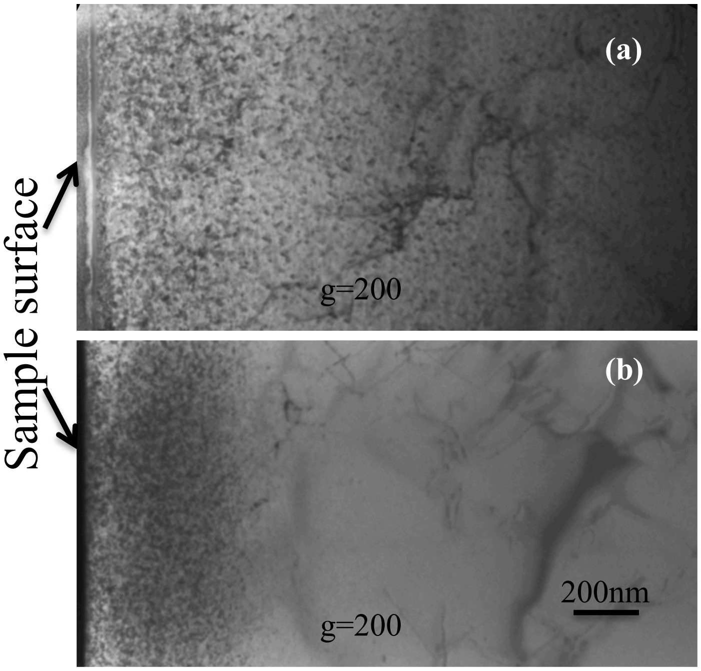

Supplementary Figure S2. Bright-field TEM images of a NiCo sample before (a) and after (b) flash polishing following the lift-out procedure with a standard FIB technique, g=[200]. The sample was initially irradiated by a 3 MeV Au ion beam to 2×10^{13} /cm^2. As demonstrated in (a), FIB polishing introduced a lot of "black dot damages" in the TEM specimen, most of which were removed by flash polishing as shown in (b).

Supplementary Video S1. Two k-ART simulations were obtained by performing simulations of a 10 keV displacement cascade in Ni at 300 K (a) and 900 K (b). Notably, small vacancy clusters (di-vacancies, tri-vacancies) were found to be much more mobile than single vacancies.

Supplementary: Stress study. We have performed 4-circle X-ray diffraction (XRD) measurements at symmetric and asymmetric diffraction peaks of (111), (002) and (113), respectively. An irradiation-induced peak broadening is observed. As compared to the full width at half maximum (FWHM) of the omega-scan through the 002 peak of ~0.1° and 0.05° for the undamaged crystals, a peak width of ~0.3° and 0.4° is identified in the irradiated NiCo and NiFe samples, respectively. The lattice in these irradiated regions, however, remains fcc phase aligned with the underlying undamaged crystal. Moreover, we observe a broadening in the 2-theta direction, which may correspond to existence of extended defects (interstitial loops) and the expected non-uniform distribution of the Au substitution at this high fluence (1.1×10^{17} /cm^2). Substitutional occupation of implanted Au ions is confirmed from ion channeling measurements on the same irradiated samples. We deduce from the XRD measurements an average lattice parameter expansion to 3.561 Å and 3.608 Å for NiCo and NiFe, as compared to the 3.533 Å and 3.589 Å for the corresponding undamaged crystals. The resulting range of 5% - 8% is in agreement with both the Au profile predicted from SRIM and measured from ion channeling technique.
